# Vaccine-linked chemotherapy induces IL-17 production and reduces cardiac pathology during acute *Trypanosoma cruzi* infection

**DOI:** 10.1038/s41598-021-82930-w

**Published:** 2021-02-05

**Authors:** Julio V. Cruz-Chan, Liliana E. Villanueva-Lizama, Leroy Versteeg, Ashish Damania, Maria José Villar, Cristina González-López, Brian Keegan, Jeroen Pollet, Fabian Gusovsky, Peter J. Hotez, Maria Elena Bottazzi, Kathryn M. Jones

**Affiliations:** 1 Texas Children’s Hospital Center for Vaccine Development, Department of Pediatrics, Baylor College of Medicine, Houston, TX USA; 2 Department of Molecular Virology and Microbiology, Baylor College of Medicine, Houston, TX USA; 3 Global Health Research, Eisai, Inc., Cambridge, MA, USA; 4 James A. Baker III Institute for Public Policy, Rice University, Houston, TX USA; 5 Laboratorio de Parasitología, Centro de Investigaciones Regionales “Dr. Hideyo Noguchi”, Universidad Autónoma de Yucatán, Mérida, Mexico; 6 Cell Biology and Immunology Group, Wageningen University & Research, De Elst 1, 6708 WD Wageningen, The Netherlands

**Keywords:** Immunology, Microbiology, Cardiovascular diseases, Infectious diseases

## Abstract

Chagas disease resulting from *Trypanosoma cruzi* infection leads to a silent, long-lasting chronic neglected tropical disease affecting the poorest and underserved populations around the world. Antiparasitic treatment with benznidazole does not prevent disease progression or death in patients with established cardiac disease. Our consortium is developing a therapeutic vaccine based on the *T. cruzi* flagellar—derived antigen Tc24-C4 formulated with a Toll-like receptor 4 agonist adjuvant, to complement existing chemotherapy and improve treatment efficacy. Here we demonstrate that therapeutic treatment of acutely infected mice with a reduced dose of benznidazole concurrently with vaccine treatment – also known as “vaccine-linked chemotherapy”—induced a T_H_17 like immune response, with significantly increased production of antigen specific IL-17A, IL-23 and IL-22, and CD8 + T lymphocytes, as well as significantly increased *T. cruzi* specific IFNγ-producing CD4 + T lymphocytes. Significantly reduced cardiac inflammation, fibrosis, and parasite burdens and improved survival were achieved by vaccine-linked chemotherapy and individual treatments. Importantly, low dose treatments were comparably efficacious to high dose treatments, demonstrating potential dose sparing effects. We conclude that through induction of T_H_17 immune responses vaccine-linked chemotherapeutic strategies could bridge the tolerability and efficacy gaps of current drug treatment in Chagasic patients.

## Introduction

*Trypanosoma cruzi* (*T. cruzi*) is a flagellated protozoan parasite described a century ago by the clinician Carlos Chagas, who proved it caused American trypanosomiasis, also known as Chagas’ disease. Chagas disease is a neglected tropical disease that afflicts 6–7 million people in 21 countries of the Americas^1,2^, with recent globalization to Europe, Japan, Australia, and elsewhere^3^. Upon infection, the parasite can invade cardiac tissue leading to clinical disease, including an asymptomatic acute phase in most cases, and later a chronic phase characterized by cardiomyopathy in the 30–40% of patients^4^. Clinical management of individual symptomatic patients can cost upwards of $11,000 annually, resulting in an annual burden of $627.46 million in health-care costs^5^. In North America an estimated 300,000 individuals are living with the illness in the United States^6^, while a recent study estimated 4 million cases in Mexico, which could represent a substantial percentage of the total cases at the Americas^7^. Historical evidence of vectorial transmission has been reported from the US since the 1930s and recently, in 2015, autochthonous cases have been reported in some southern states, more specifically in Texas^8,9^. Additionally, recent studies of congenital transmission have revealed significant seroprevalence in both Mexico (6.6%), Argentina (6.3%), and other Latin American countries^10^.

The clinical form of Chagas disease known as Chronic Chagas cardiomyopathy (CCC) is characterized by chronic inflammation and progressive fibrosis in the heart^11,12^. Over time the fibrotic tissue can disrupt the cardiac electrical conduction system leading to arrhythmias, sudden death, or progressive heart failure^13^. Importantly, in CCC patients, myocardial fibrosis correlates with disease severity and has been shown to be an independent predictor of adverse outcomes^14^. In addition to the direct role of fibrosis in CCC, several studies in human and pre-clinical studies have partially defined the correlation between the inflammatory immune response and disease severity. For instance, a study in chronically infected patients has shown that a pro-inflammatory parasite specific immune response with higher levels of IFNγ correlates with severe disease, whereas a balanced immune response with both IFNγ and the regulatory cytokine IL-10 correlates with less severe or no clinical disease^15^. Further studies have identified the T_H_17 immune response, driven by the cytokine IL-17A, as a direct mediator of protection against *T. cruzi*-related mortality^16^. Understanding the correlation between a balanced immune response and less severe clinical outcomes has led to harnessing its potential in the development of therapeutics and vaccines against Chagas disease.

The two antiparasitic drugs approved for treatment of *T. cruzi* infection are benznidazole (BZN) and nifurtimox, but both drugs have major failures in tolerability and efficacy. The current recommended BZN treatment dose for asymptomatic chronic adult patients is 5–10 mg/kg per day for 60 days^17–19^. However, up to 40% of patients discontinue treatment due to severe side effects, including dermatologic manifestations, digestive disturbances, and neurological disorders^17,20^. Thus, there is great interest in improving drug tolerability, including reduced dose and combination drug treatments^21,22^. Additionally, only BZN is licensed for treatment of Chagas disease in the US, but it has not been shown to be effective during the chronic phase of disease^23^. The Benznidazole Evaluation for Interrupting Trypanosomiasis (BENEFIT) trial, the largest study of Chagasic patients to evaluate BZN treatment, failed to ameliorate prevent disease progression and cardiac death compared to placebo^24,25^. Thus, BZN is useful for the acute and early chronic stages of Chagas disease, but it does not improve survival in patients with CCC.

We are developing a therapeutic vaccine to complement current drug treatments by reducing cardiac parasite burdens, and parasite induced inflammation and fibrosis. Our lead candidate vaccine is based on the *T. cruzi* derived flagellar antigen Tc24-C4 as a 24 kDa recombinant protein which has been genetically engineered to alter four cysteine residues in order to reduce intermolecular disulfide bond formation and aggregation during production^26^. Early protein and DNA based vaccines containing the Tc24 antigen reduced parasite burdens, cardiac inflammation and severity in the electrocardiographic alterations in rodent and dog models of infection^27–31^. More recently, recombinant protein based Tc24 vaccines formulated with the TLR4 agonist MPLA were shown to reduce cardiac parasite burdens and improved survival during acute infection when used as a preventative vaccine^32^. When used therapeutically in acutely infected mice, vaccines containing recombinant Tc24 protein encapsulated in PLGA nanoparticles, or dendritic cells loaded with both Tc24 protein and Tc24 containing adenovirus vector, reduced parasite burdens and cardiac pathology while inducing both pro-inflammatory (IFNγ) and anti-inflammatory (IL-4) cytokines^33,34^. Importantly, we recently demonstrated that therapeutic vaccination of chronically infected mice resulted in significantly reduced cardiac fibrosis, while inducing significantly increased antigen specific IFNγ production^35^. Together, these data clearly demonstrate efficacy of Tc24 based vaccines via immune mediated control of both parasite burdens and cardiac pathology.

We have also examined the potential for improved BZN efficacy by linking anti-parasitic drug therapy to immunotherapy with our Tc24-C4 recombinant protein vaccine, known as “vaccine-linked chemotherapy”. In our initial proof-of-concept study using this strategy, acutely infected BALB/c mice were treated with a reduced BZN dose of 25 mg/kg daily for 7 days starting at day 7 post-infection followed by vaccination with a stable emulsion of Tc24-C4 and the TLR4 agonist E6020 on days 17 and 24. This regimen significantly reduced parasite burdens and cardiac inflammation, and significantly increased antigen specific CD8 + T lymphocytes, as well as the cytokines IFNγ, IL-4, and IL-10, indicating a balanced T_H_1/T_H_2 immune response^36^. This was a key study demonstrating that reduced dose benznidazole treatment can be improved by the addition of vaccine immunotherapy. Therefore, we sought to further optimize this vaccine-linked chemotherapy strategy by evaluating high- and low-dose vaccine formulations and initiating BZN and immunotherapy concurrently. Beyond the balanced immune responses found previously, here we report that vaccine-linked chemotherapy also induces a T_H_17 like response. Recently it has been demonstrated in mouse models that the IL-17A cytokine family is sustains CD8 + T cell immunity to *T. cruzi ,* which helps to clear the infection by eliciting stronger cell-mediated cytotoxic responses^37^. Additionally, IL-17A regulates influx of inflammatory cells into cardiac tissue in a mouse model of *T. cruzi* infection^38^. Therefore, our findings will help us to understand a new role for T_H_17 responses added to a balanced T_H_1/ T_H_2 parasite-specific immune response as a means to understand the immunological basis for controlling both parasite burdens and cardiac pathology.

## Results

### Concurrent Tc24-C4 vaccine with BZN increases survival and reduces parasite burdens

**Figure 1:**
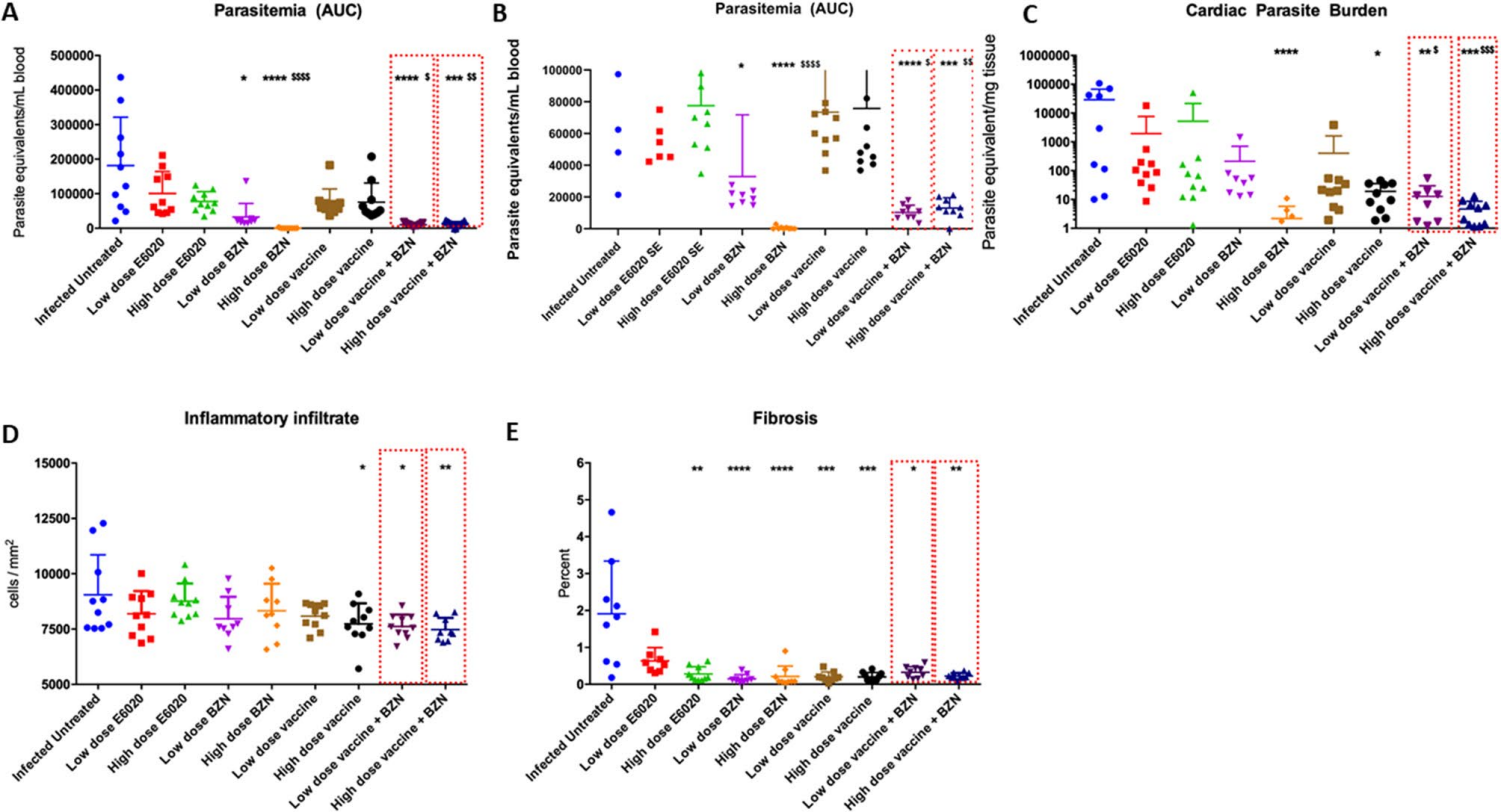
Vaccine-linked chemotherapy efficacy by reducing parasitemia, cardiac parasite burden, inflammatory infiltrate and fibrosis. (**A**) The parasitemia represented as Area Under Curve (AUC) determined along 53 days post-infection by qPCR. (**B**) Adjusted scale of AUC showing differences between treatment groups. (**C**) Cardiac parasite burden was determined by qPCR at 53 dpi. (**D**) Inflammatory infiltrate cells were determined by 5 representative images from each mouse analyzed by FIJI software from cardiac tissue slides using hematoxylin/eosin staining. (**E**) Fibrosis percentage was determined by 5 representative images from each mouse analyzed by FIJI software from cardiac tissue slides using trichrome staining. *, *P* ≤ 0.05; **, *P* ≤ 0.01; ***, *P* ≤ 0.001 indicate difference when comparing groups to the infected untreated control*.* $, *P* ≤ 0.05; $$, *P* ≤ 0.01; $$$, *P* ≤ 0.001; $$$$, *P* ≤ 0.0001 indicate when comparing groups to the low-dose BZN. &, *P* ≤ 0.05; &&, *P* ≤ 0.01 when comparing groups to the low-dose E6020.

To evaluate protection given by the concurrent Tc24-C4 vaccine in combination with BZN, we monitored survival and collected blood to measure parasitemia until 53 days of infection (53dpi). As expected, infected untreated control mice exhibited an acute lethal infection with only 40% survival at 53 dpi. Each treated-experimental group had significantly increased survival compared with the untreated control group (Table 1). Importantly, both groups that received the concurrent vaccine with BZN, using either a high or low-dose vaccine, had similar survival (100%) as the control groups treated with the curative or low-dose of BZN. Reduced but still high survival rates (90%) were observed in mice treated with both high and low doses of E6020-SE alone. The group treated with the low-dose vaccine alone had 80% survival rate, which did not reach statistical significance compared with the infected untreated control group (Table 1). Parasitemia, as measured by the area under the curve (AUC) was also significantly reduced by both low and high dose vaccine + BZN treatment as well as low dose BZN and high dose BZN alone (Fig. 1A,B). Importantly, both low and high dose vaccine + BZN groups had significantly lower AUC when compared to low dose BZN alone, suggesting a synergistic effect of the combined treatment (Fig. 1A,B). Further, both low and high dose vaccine + BZN groups showed the same levels of protection as the group receiving high dose BZN. While mice vaccinated with either a high or low-dose of the vaccine alone or E6020-SE alone showed a trend to a lower parasitemia compared to infected untreated controls, these reductions did not reach statistical significance (Fig. 1A,B). Supporting the reduced parasitemia, mice treated with both low and high dose vaccine + BZN had significantly lower cardiac parasite burdens compared to the infected untreated mice, and compared to mice treated with low dose BZN alone (Fig. 1C). In contrast, the groups treated with low-dose of the vaccine, low-dose BZN, high and low-dose E6020 failed to reduce the cardiac parasite burden (Fig. 1C). Our results clearly showed that vaccine-linked chemotherapy was able to protect mice from dying during acute infection by decreasing parasitemia levels.

**Table 1:** Survival to *T. cruzi* acute infection.

Group	Percent Survival (%)	P value
Infected Untreated	40	N/A
Low dose E6020	90	0.0203*
High dose E6020	90	0.0234*
Low dose BZN	100	0.0039**
High dose BZN (curative)	100	0.0039**
Low dose vaccine	80	0.0598
High dose vaccine	100	0.0039**
Low dose vaccine + BZN	100	0.0039**
High dose vaccine + BZN	100	0.0039**

Mice were infected with 500 trypomastigotes of *T. cruzi* and followed up daily for up to 53 days post-infection. Percentages of survival were analyzed using Log-rank (Mantel-Cox) test comparing treated groups vs infected untreated group and statistical differences were considered when *P* values * *P* ≤ 0.05, ** *P* ≤ 0.005.

### Concurrent Tc24-C4 vaccine with BZN protects mice against acute cardiac inflammation and fibrosis

We also evaluated the potential of vaccine-linked chemotherapy to protect against tissue damage caused by *T. cruzi* acute infection by measuring cardiac inflammatory cell inflammation and fibrosis. The percentage of the inflammatory cell infiltrate in the heart of mice treated with either a high- or low-dose of vaccine + BZN, as well as high dose vaccine alone, was significantly lower compared with the infected untreated control group (Fig. 1D). Concurrently, fibrosis was significantly reduced by all experimental treatments except low-dose E6020 , when compared to infected untreated control mice (Fig. 1E). Together, these results suggest that vaccine-linked chemotherapy also protects against cardiac damage caused by acute *T. cruzi* acute infection.

### Concurrent Tc24-C4 vaccine with BZN induces a robust T cell-mediated immune response

**Figure 2:**
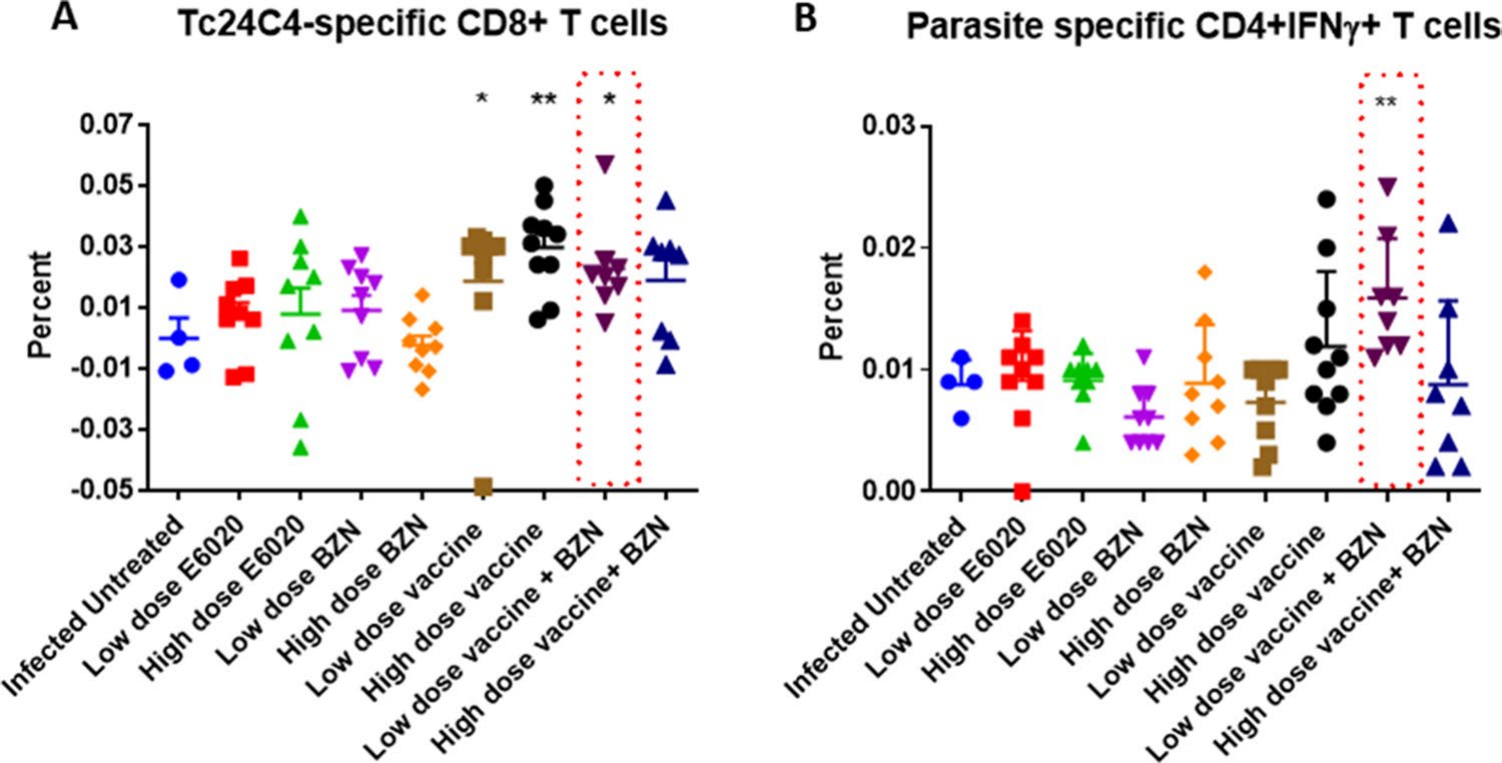
Cellular immune response against *T. cruzi antigens*. Spleen cell cultures were stimulated for 96 h and stained with fluorescent antibodies for further flow cytometry analysis. (**A**) Frequency of Tc24-C4 antigen-specific CD8 + T cells for the different experimental groups. (**B**) Frequency of total parasite antigen-specific CD4 + IFNγ + T cells for the experimental groups. *P ≤ 0.05, ** *P* ≤ 0.005 indicate difference when comparing groups to the infected untreated control.

To evaluate the participation T cell subsets induced by vaccine-linked chemotherapy in the control of acute *T. cruzi* infection spleen cells harvested from survivor mice were stimulated in vitro with Tc24-C4 protein or *T. cruzi* parasite lysate, stained with fluorescent antibodies and analyzed by flow cytometry, as described. Results indicated that high-dose vaccine + BZN, high-dose vaccine alone, and low-dose vaccine alone induced a significantly higher percentage of Tc24-C4 specific CD8 + T cells compared to infected untreated control mice (Fig. 2A). Moreover, mice treated with low-dose vaccine + BZN had a significantly higher percentage of parasite-specific CD4 + IFNγ producing-T cells compared to infected untreated control mice (Fig. 2B). There were no significant differences in IFNγ producing- CD8 + T cells between the infected untreated mice and any of the treatment groups (data not shown). Thus, our data demonstrate that our vaccine, alone or in combination with low dose BZN treatment, induces a strong antigen-specific T cell response.

### Concurrent Tc24-C4 vaccine with BZN elicited a robust Tc24-C4-specific T_H_17 like immune response

**Figure 3:**
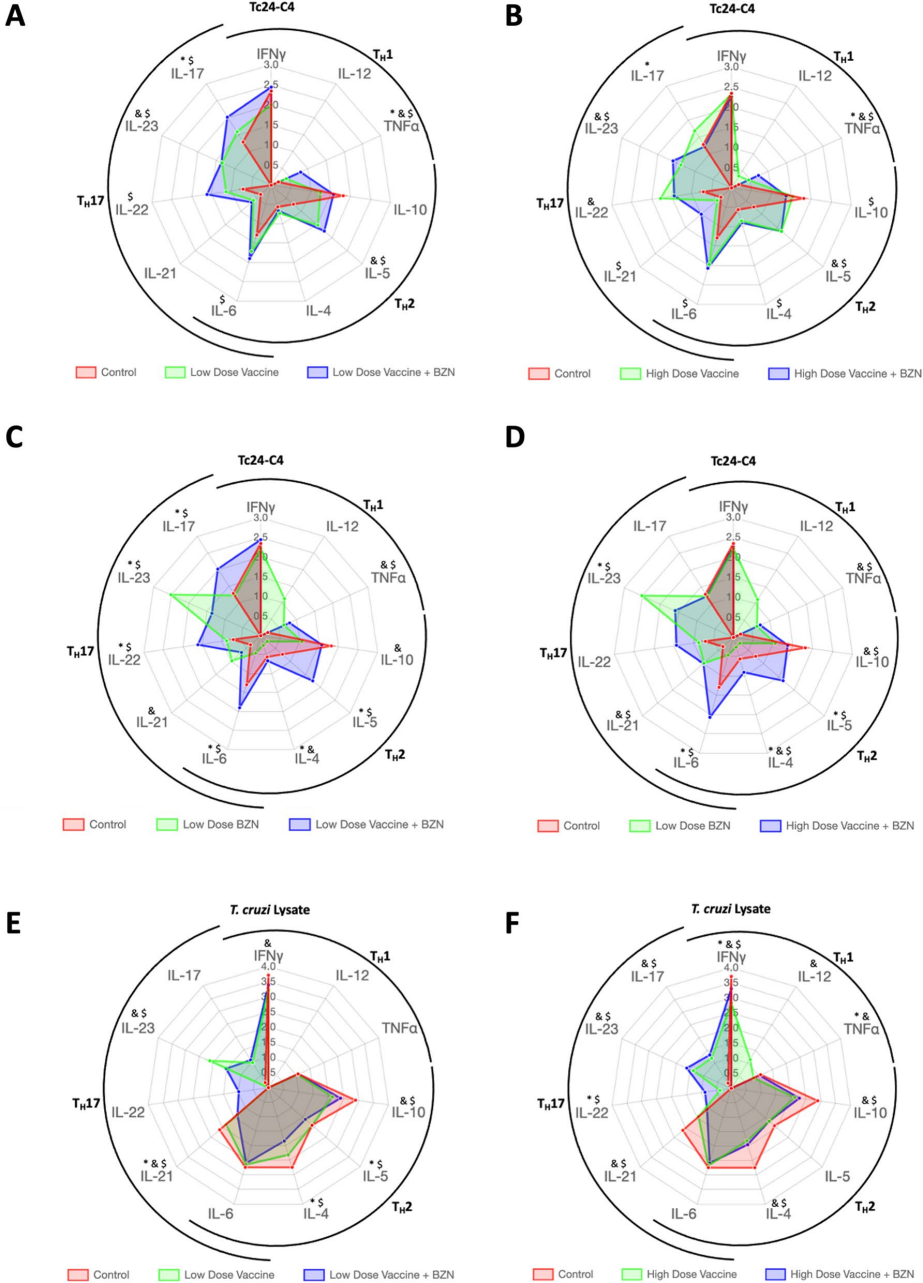
Radar plots showing the T_H_1/T_H_2/T_H_17 like soluble cytokine profile. Cytokines were measured in supernatants of splenocyte cultures after 96 h of restimulation with Tc24-C4 protein (**A**–**D**) or *T. cruzi* Lysate (**E**–**F**) using a Luminex assay as described. Low dose vaccine + BZN (25 µg Tc24-C4/5 µg E6020 SE + 25 mg/kg BZN) significantly increased Tc24-C4 specific IL-17, IL-22, IL-23, IL-6, TNFα and IL-5 production when compared to infected untreated controls, and significantly increased IL-17 and TNFα when compared to low dose vaccine alone (**A**). In comparison, high dose vaccine + BZN induced significantly increased Tc24-C4 specific IL-23, IL-21, IL-6, IL-4, IL-5, IL-10 and TNFα when compared to infected untreated control mice, and decreased IL-17 and TNFα when compared to high dose vaccine alone (**B**) Similarly, low dose vaccine + BZN induced significantly increased Tc24-C4 specific IL-17, IL-22, IL-23, IL-6, TNFα and IL-5 production when compared to low dose BZN (**C**). In comparison , high dose vaccine + BZN induced significantly increased Tc24-C4 specific IL-23, IL-21, IL-6, IL-4, IL-5, IL-10 and TNFα when compared to low dose BZN (**D**). Low dose vaccine + BZN also induced significantly increased total parasite IL-21, IL-23, IL-10, IL-5, and IL-4 when compared to infected untreated controls, and increased IL-21, IL-4 and IL-5 compared to low dose vaccine alone (**E**). Low dose vaccine alone induced significantly increased IFNγ, IL-21, IL-23 and IL-10 compared to infected untreated controls (**E**). Similarly, high dose vaccine + BZN also induced significantly increased total parasite IFNγ, IL-10, IL-4, IL-21, Il-22, IL-23 and IL-17 compared to infected untreated controls, and increased IFNγ, IL-12, TNFα, IL-10, IL-4, IL-21, IL-23, and IL-17 when compared to high dose vaccine alone (**F**). High dose vaccine induced significantly increased IFNγ, TNFα and IL-22 compared to infected untreated mice. ^$^Indicates a statistically significant difference (*P* value ≤ 0.05) when comparing low or high dose vaccine + BZN to infected untreated control mice (**A**,**B**,**E**,**F**), or when comparing low or high dose vaccine + BZN to low dose BZN alone (**C** and **D**). *Indicates a statistically significant difference (*P* value ≤ 0.05) when comparing low or high dose vaccine + BZN to vaccine alone (**A** and **B**), or when comparing low or high dose vaccine + BZN to low dose BZN alone (**C** and **D**). ^&^Indicates a statistically significant difference (*P* value ≤ 0.05) when comparing low or high dose vaccine alone to infected untreated control mice (**A**,**B**,**E**,**F**), or when comparing low dose BZN to infected untreated controls (**C** and **D**).

We previously showed that vaccine-linked chemotherapy induces a balanced antigen specific T_H_1/T_H_2 cytokine profile in acutely infected mice^36^. Here we further investigated the antigen specific T_H_17 cytokine profile induced by vaccine-linked chemotherapy. We found that mice treated with a low-dose of vaccine + BZN had significantly higher levels of Tc24-C4 specific IL-17, IL-22, IL-23, IL-6, TNFα and IL-5 production when compared to infected untreated controls, and significantly increased IL-17 and TNFα when compared to low dose vaccine alone, suggesting increased T_H_17 like responses in addition to T_H_1 like and T_H_2 like responses (Fig. 3A). Likewise, mice receiving a low-dose of the vaccine alone had significantly higher levels of IL-23, TNF-α, IL-5 and a trend toward high levels of IL-17A and IL-22. High-dose vaccine + BZN treatment induced significantly increased Tc24-C4 specific IL-23, IL-21, IL-6, IL-4, IL-5, IL-10 and TNFα when compared to infected untreated control mice, and increased IL-17 and TNFα when compared to high dose vaccine alone (Fig. 3B). In contrast, high dose vaccine + BZN induced lower levels of IL-17 and IL-22 compered to vaccine alone (Fig. 3B). Described previously as a regulatory cytokine, IL-10 was significantly increased in infected untreated mice compared to mice treated with high-dose vaccine + BZN (Fig. 3B). Additionally, mice treated with low dose vaccine + BZN showed significantly higher levels of T_H_17 (IL-17A, IL-22) and T_H_2 (IL-4, IL-5, IL-10) when compared to mice treated with low-dose BZN alone (Fig. 3C), while mice treated with high dose vaccine + BZN showed increased IL-4, IL-5, and IL-6 when compared to low dose BZN alone (Fig. 3D). Conversely, the cytokine IL-23 was significantly higher in the group treated with low-dose BZN alone compared to vaccine + BZN (Fig. 3C–D). Levels of IFNγ and TNF-α were found similar between mice treated with either low or high-dose vaccine + BZN and low-dose BZN alone (Fig. 3C–D) As expected, treatment with low-dose BZN alone did not induce Tc24-C4 specific T_H_2 cytokine release (Fig. 3C–D).

### Concurrent Tc24-C4 vaccine with BZN induced a robust total parasite T_H_17 cytokine response

We also evaluated and compared the cytokine release response against *T. cruzi* total parasite antigens. As expected, infected untreated mice showed a T_H_2 biased cytokine profile with higher levels of IL-10, IL-5, IL-4 in contrast with IL-17A, IL-23, IL-22 and IL-12p70 levels that were undetectable (Fig. 3E–F). Also, higher levels of IFNγ were observed in the infected untreated group compared to all other treatment groups (Fig. 3E–F). Mice treated with a low-dose vaccine + BZN showed significantly lower levels of T_H_2 cytokines (IL-4, IL-5, and IL-10) as well as higher levels of IL-17A, IL-23 and IL-22 compared to the infected untreated group (Fig. 3E). Interestingly, mice treated with high-dose vaccine + BZN showed a T_H_17 like immune response with significantly higher levels of IL-17A, IL-23 and IL-22 and significantly lower levels of IL-10 and IL-4 (Fig. 3E). Radars graphs showing the total parasite-specific cytokine patterns from the groups treated with low or high-dose E6020-SE alone showed an overall T_H_2 biased profile with increased IL-4, IL-5 and IL-10 cytokines, but with significantly lower levels compared to infected untreated mice (Supplementary Fig. 1A). Tc24-C4 specific IL-22 was significantly reduced and IL-23 was significantly increased in mice treated with high-dose E6020-SE alone compared to infected untreated mice (Supplementary Fig. 1B). As expected, the groups treated with E6020 alone had higher levels of soluble IFNγ compared to mice infected untreated. Nevertheless, the T_H_17 profile in those mice was slightly increased (IL-21 and IL-22 compared to infected untreated mice (Supplementary Fig. 1).

### The T_H_17 like cytokine response strongly correlated with reduced parasite burdens

**Figure 4:**
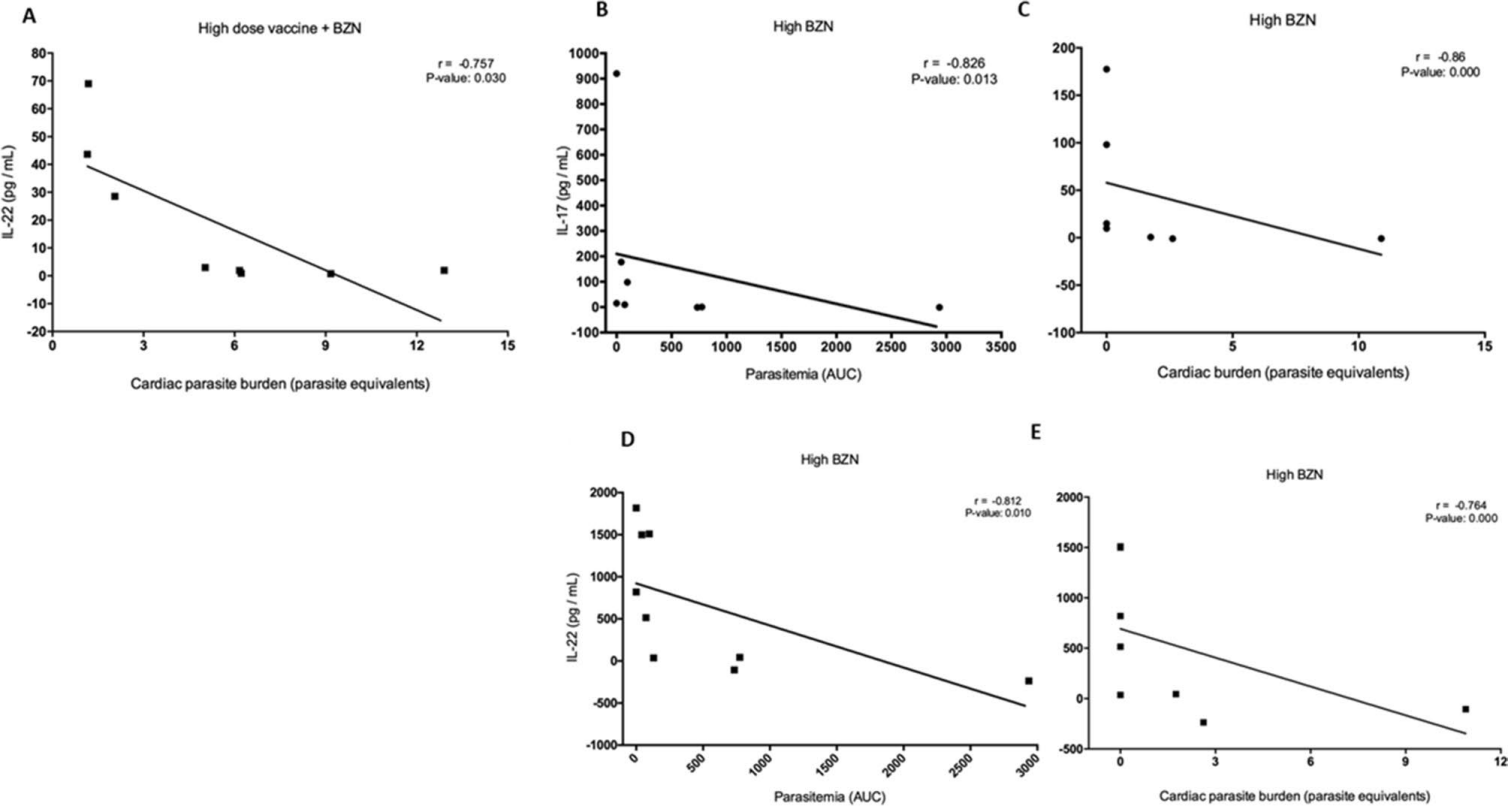
Correlation analyses of cytokines and parasite burdens. Mice were treated either concurrently with high dose vaccine + BZN (100 μg Tc24-C4/25 μg E6020-SE + 25 mg/Kg BZN), or a high dose of BZN (100 mg/kg BZN) starting 7 days post-infection as described. Cardiac parasite burdens were determined by qPCR at 53 days of infection, and parasitemia was measured twice weekly until 53 days of infection by qPCR as described. Cytokine levels were measured from the supernatant of splenocytes restimulated with *T. cruzi* lysate by Luminex assay as described. Levels of IL-22 were significantly negatively correlated with cardiac parasite burdens in mice treated with high dose vaccine + BZN (**A**); Levels of IL-17 were significantly negatively correlated with parasitemia (**B**) and cardiac parasite burdens (**C**) in mice treated with high dose BZN. Similarly, levels of IL-22 were significantly negatively correlated with parasitemia (**D**) and cardiac parasite burdens (**E**) in mice treated with high dose BZN. P ≤ 0.05 were considered statistically significant.

To investigate if parasite burdens were associated with specific cytokines, we performed a correlation analysis comparing parasitemia and cardiac parasite burden with the T_H_17 cytokines IL-17A, IL-21, IL-22, and IL-23. Importantly, a significant negative correlation (*r* = -0.75, *P-*value = 0.030) was found between cardiac parasite burdens and IL-22 in mice treated with the high-dose vaccine + BZN (Fig. 4A). Further, significant negative correlations were found in mice treated with the curative dose of BZN between IL-17A and both parasitemia and cardiac parasite burden (r = -0.826, *P*-value *Spearman* 0.013; r = -0.86, P < 0.0001 respectively) (Fig. 4B and C, respectively), as well as between the cytokine IL-22 and both parasitemia and cardiac parasite burden (r = -0.812, *P*-value *Spearman* 0.010; r = -0.764, P < 0.0001 respectively) (Fig. 4D and E, respectively). To begin to explore the correlation between CD4 + and CD8 + cytokine producing cells and secreted cytokines from cultured splenocytes, we performed a correlation analysis between the percent of CD4 + IFNγ + cells or CD8 + IFNγ + cells measured by flow cytometry and individual secreted cytokines measured by Luminex. We found that mice treated with low dose vaccine + BZN had a significant negative correlation with secreted TNFα (Supplementary Fig. 2A). In contrast, mice treated with high dose vaccine + BZN had a significant positive correlation with secreted TNFα (Supplementary Fig. 2B). No other significant correlations were identified between specific secreted cytokines or cytokine producing T cells and cardiac parasites or parasitemia.

## Discussion

Vaccine linked-chemotherapy is a promising strategy combining the beneficial effects of both vaccine immunotherapy and an antiparasitic drug to control acute *T. cruzi* infection^36^. In this study, we demonstrated that either high or low-dose vaccine given concurrently with low-dose BZN significantly reduced cardiac parasite burdens, parasitemia, cardiac inflammatory infiltrate and cardiac fibrosis in acutely infected mice. Further, concurrent vaccine + BZN treatment induced antigen-specific cytokines indicative of T_H_17 like immune responses, in addition to T_H_1 and T_H_2 like immune responses. These data confirm and expand on our prior results, where by using a sequential vaccine-linked chemotherapy strategy with treatment initiated on 7 days post infection, parasitemia was reduced by 24% and the secreted cytokine profile from splenocytes suggested a balanced T_H_1/T_H_2 immune response^36^. Here we improved on that result, achieving a 98.73% reduction in parasitemia by concurrently administering vaccines and low-dose of BZN starting 7 days post-infection. We recently reported on the partially protective effect of TLR4 agonist adjuvant in acutely infected mice mediated in part by reduction of tissue parasite burdens^39^. Here we confirmed our prior results showing partial efficacy of individual treatments with benznidazole, vaccine, or the TLR4 agonist adjuvant E6020 alone as evidenced by significantly increased survival, reduced parasite burdens and reduced fibrosis. Importantly in this study only vaccine + BZN at both high and low doses and high dose vaccine alone significantly reduced cardiac inflammatory infiltrate, suggesting that both vaccine and low dose benznidazole are needed to achieve maximal synergistic effects. However, due to the partial protection afforded by each individual treatment additional studies combining specific components, such as E6020 SE + BZN, would be required to further define the relative contribution and synergism of the Tc24 C4 antigen, the TRL4 agonist adjuvant, and benznidazole on vaccine-linked chemotherapy efficacy. We also showed that the effects of high and low dose vaccine + BZN on parasite burdens and tissue pathology were roughly equivalent, indicating that the low dose vaccine + BZN could be advanced in further testing as a dose sparing strategy.

Parasite persistence has been demonstrated to drive the persistent low grade inflammation and post-inflammatory fibrosis of symptomatic chronic Chagas disease, which results in pathologic cardiac remodeling, and dysfunctional electrical conduction^13,40,41^. Indeed, myocardial fibrosis in humans determined by non-invasive techniques like magnetic resonance imaging was correlated to severe heart disease^14,42^. Recently, we also showed in a mouse model of chronic infection that cardiac fibrosis correlated with increased cardiac strain on MRI, and increased serum levels of the pro-fibrotic biomarkers TGFβ, platelet derived growth factor-D (PDGF-D), and connective tissue growth factor (CTGF)^43^. Our group has previously shown that therapeutic vaccination with a high dose prototype vaccine (100 µg Tc24 + 25 µg E6020-SE) significantly reduced cardiac fibrosis in chronically infected mice^35^. Building on these findings, ongoing studies are evaluating the effect of low dose vaccine-linked chemotherapy in a chronic infection model as a dose sparing strategy to improve cardiac health. Evaluating cardiac function using non-invasive cardiac imaging, such as echocardiography, as well as evaluating general animal health in the ongoing chronic studies will be key to determine potential clinical improvement, and may suggest specific parameter for monitoring clinical outcomes in future human clinical trials.

We measured the major cytokines representing T_H_17, T_H_1, and T_H_2 immune responses to define their participation in the protection against *T. cruzi* infection conferred by vaccine + BZN. Vaccine-linked chemotherapy induced a T_H_17 like profile, with significantly higher levels of IL-17, IL-22, and IL-23 compared to infected untreated controls. Further, low dose vaccine + BZN induced increased IL-17A secretion when compared to low dose vaccine alone**.** However, high dose vaccine + BZN induced IL-17A levels that were lower than high dose vaccine alone. Recent findings have shown that the cytokine IL-17A in particular is critical for eliciting a T_H_17 immune response, and is necessary for *T. cruzi* host protection^16^. IL-17A has been shown to regulate cardiac inflammation in experimentally infected animals mediated by neutrophil derived IL-10, and IL-17RA signaling is necessary for survival of parasite specific CD8 + T cells^37,38,44^. Further, it has been confirmed in Chagasic patients that increased IL-17A expression and increased CD4 + IL-17A producing cells correlate with less severe cardiac disease^45,46^. Thus, we conclude that the decreased cardiac inflammatory infiltrate and parasite burdens and increased antigen specific CD8 + cells is mediated in part by increased IL-17 induced by vaccine-linked chemotherapy. The interesting finding of reduced IL-17 secretion in the high dose vaccine + BZN treatment compared to high dose vaccine alone may be due to further reductions in parasite burdens from the higher doses which may in turn reduce the parasite stimulus for increased IL-17. Additional studies exploring different doses of vaccine and BZN would be necessary to elucidate this.

The role of the cytokine IL-23 in *T. cruzi* infection is poorly described, however, its participation in bacterial infections such as *Listeria monocytogenes* is critical for neutrophil recruitment mediated also by IL-17 receptor A (IL-17RA) in an IFNγ independent manner^47^. Moreover, both cytokines (IL-17A/IL-23) have been shown to have a protective role during other vacuole-bound bacteria and *Toxoplasma gondii* infections^48,49^. Thus, our findings indicate that resistance to *T. cruzi* infection might be associated to IL-17A/IL-23 axis. Another T_H_17 cytokine that could be associated with protection against *T. cruzi* is IL-22. Besides its protective role in *T. cruzi* immunity being not well elucidated, efforts have been made to define its association with parasite genetic diversity^50^ or pathogenicity^51^. Results from the latter study reported that lower total serum levels of IL-22 in mice were associated with intestinal damage during chronic *T. cruzi*-infection. Potentially, the consistently increased levels of either Tc24-C4 specific or total *T. cruzi* parasite-specific IL-22 from mice treated with vaccine + BZN, in sharp contrast with undetectable levels from infected untreated mice, would be strongly related to protection. Additionally, increased IL-22 levels from mice therapeutically treated with vaccine + BZN significantly correlated with decreased *T. cruzi* parasite burdens in cardiac tissue. Even though associating a single cytokine with clinical outcomes might be difficult due to their pleiotropic expression, these data suggest IL-22 may be a useful biomarker for treatment efficacy^50^. Based on previous studies in vitro*,* a T_H_17 immune profile seems to be paramount for control of acute *T. cruzi* infection, likely through NADPH oxidase mechanisms with the collaboration of IL-21 and IL-22^16^.

In addition to demonstrating the T_H_17 like responses induced by vaccine-linked chemotherapy, we also confirmed that this strategy induces T_H_1 and T_H_2 like immune responses. The low dose vaccine + BZN treatment induced significantly increased antigen specific CD8 + cells, total parasite CD4 + IFNγ + cells, and the cytokines TNFα, and IL-5 suggesting a mixed T_H_1/T_H_2 response. The T_H_1 cytokines TNFα, IL-12, and IFNγ, are well-known to be necessary for activation of antigen-presenting cells (APCs), and stimulation of inducible Nitric Oxide Synthase (iNOS), a critical killing mechanism for intracellular *T. cruzi* parasites^52–55^. In addition, CD8 + T lymphocytes are essential for control of *T. cruzi* infection, and both vaccines and benznidazole treatment have been shown to induce protective CD8 + T lymphocyte populations^56,57^. Together, these data suggest that the mechanism of protection afforded by our vaccine-linked chemotherapy strategy can also be attributed to the induction of T_H_1 and T_H_2 driven effector mechanisms.

A discordant finding in this study was that neither antigen-specific nor total parasite IFNγ release was significantly increased in our vaccine + BZN concurrent treatments. IFNγ plays an essential role in the recruitment T cells in the initial stage of *T. cruzi* infection and is critical for protective immunity in both mice and humans^34,35,58,59^. However, overexpression leads to undesirable inflammatory effects^60^. IL-10 has been described as a T_H_2 regulatory cytokine that inhibits MHC class II expression and excessive production of IFNγ and TNF-α^61^. Here we did not observe significantly increased IL-10 secretion induced by vaccine alone or vaccine + BZN when compared to infected untreated mice. In fact, IL-10 secretion appeared to be slightly reduced, although this decrease was not statistically significant. One explanation for this result is that vaccine-linked chemotherapy controlled both parasite burdens and cardiac inflammation such that by 53 days post infection, the immune response is reflective of a balanced, healing response. Another explanation is that in this model, the antigen specific T_H_17 immune response may contribute more to controlling cardiac pathology compared to IL-10. Still another possibility is that under some as yet undefined circumstance in mice or related to dose and formulation, the vaccine might protect through either T_H_17 or balanced T_H_1/T_H_2 pathways. Further studies are necessary to identify and characterize the specific cells involved in both parasite control and cardiac pathology, including cardiac specific lymphocytes. In contrast to IFNγ, both low and high dose vaccines, either alone or in combination with BZN treatment, did induce significantly increased levels of IL-6. IL-6 is a pleiotrophic cytokine that is essential for parasite specific immune responses and resistance to *T. cruzi* infection^62^. However, IL-6 is also known to promote fibrosis in the setting of extensive inflammation^63^. Since we observed reduced fibrosis and inflammation in our model, it is likely that the anti-inflammatory effects of the vaccine reduced the expected biologic impact of this cytokine. Future studies will further evaluate the relationship between treatment induced cytokines and pro-fibrotic biomarkers to determine their relative contribution to cardiac pathology.

In summary, we demonstrated that a concurrent vaccine-linked chemotherapy treatment strategy can achieve protection against acute *T. cruzi* infection by immune mediated control. The antigen specific cytokine profile suggests a balanced immune response, with T_H_1 like, T_H_2 like and T_H_17 like responses. The protective immune response induced by our concurrent vaccine-linked chemotherapy strategy is similar to other protective vaccines^64–66^. Further, the comparable efficacy of the low dose vaccine + BZN to the high dose vaccine + BZN indicates that there is no practical benefit to using higher doses. This work provides key proof of concept for employing multi-modal treatment options to bridge the efficacy and tolerability gaps of benznidazole treatment alone, but additional knowledge of the mechanisms of disease will assist in further optimizing treatment strategies. Ongoing studies in our lab using vaccine-linked chemotherapy in chronically infected mice are exploring the effect of this strategy on cardiac pathology, including inflammation and fibrosis, as well as cardiac function and liver health as measures of improved clinical outcomes. Additionally, defining functional differences in immune cells within the periphery, i.e., antigen specific CD8 + IFNγ + , CD4 + IFNγ, and CD4 + IL-17A + cells, will better define the immune response induced by vaccine-linked chemotherapy. Further, defining the cardiac specific inflammatory response, for example by staining for inflammatory markers by immunohistochemistry, will provide critical information about the mechanisms of disease that correlate with cardiac health. Together, these data will ultimately assist in the identification of additional targets for therapeutic intervention.

## Material and methods

### Ethics statement

All studies were approved by the Institutional Animal Care and Use Committee of Baylor College of Medicine described under assurance numbers D16-00,475 (current) and 3823–01 (previous) and were performed in strict compliance with the 8^th^ Edition of The Guide for Care and Use of Laboratory Animals^67^.

### Parasite and mice

*T. cruzi* H1 parasites, originally isolated from a human case in Yucatan, Mexico were maintained by serial passage in mice every 25 to 28 days until infection^68^. Ninety female BALB/c mice were obtained at 6-week-old from Taconic vendor (Taconic Biosciences, Inc) and housed in groups of 5 animals, with ad libitum food and water and a 12 h light/dark cycle. Mice were allowed to acclimate for one week prior to studies.

### Parasite lysate

Parasite lysates was prepared using 10^6^ epimastigotes *T. cruzi* H1 obtained from logarithm grown phase of epimastigotes cultured in LIT media at 28 °C. The parasites were disrupted in PBS containing protease inhibitor cocktail (cOmplete ultra-tablets, Roche) by three freeze–thaw cycles. Further, parasites were sonicated for 15 s at the minimum setting three times and then centrifuged at 15,000 g for 30 min. The soluble fraction was aliquoted and stored at -80 °C until use. Protein concentration was determined by BCA protein assay kit (ThermoFisher) according to manufacturer’s instructions.

### Immunotherapeutic treatment

All mice were infected with 500 blood form *T. cruzi* H1 trypomastigotes by intraperitoneal injection. Infected mice were randomly divided into nine experimental groups of 10 mice each (Table 2). The recombinant Tc24-C4 antigen was expressed and purified according to previously published work^26^. The adjuvant E6020 was dissolved in a 2% stable squalene emulsion (SE), acquired through Eisai Inc. Vaccine formulations were freshly prepared and mixed just before injection. Prepared vaccines were injected subcutaneously on day 7 and boosted at day 14 post-infection. BZN powder (ELEA Laboratory) was reconstituted in solution with 95% HPMC (0.5% hydroxypropylmethylcellulose/ 0.4% Tween 80/ 0.5% benzyl alcohol in deionized water) and 5% DMSO to a final concentration of 10 mg/mL and prepared daily, immediately before oral administration to mice. Mice receiving low-dose (25 mg/kg) daily oral BZN (BZN) were treated for 1 week from 7 days until 14 days post-infection. Mice receiving high-dose (100 mg/kg) daily oral BZN were treated for 20 days from 7 days until 26 days post-infection. Survival was recorded daily throughout the experiment and 53 days post-infection all surviving mice were humanely euthanized, and samples of heart and spleen were taken for further assays.

**Table 2:** Experimental groups.

Group	Vaccine/adjuvant	Drug
Infected Untreated	None	None
Low dose E6020	5 µg E6020 SE	None
High dose E6020	25 µg E6020 SE	None
Low dose BZN	None	25 mg/kg orally for 7 days
High dose BZN (curative)	None	100 mg/kg orally for 7 days
Low dose vaccine	25 µg Tc24-C4 + 5 µg E6020 SE	None
High dose vaccine	100 µg Tc24-C4 + 25 µg E6020 SE	None
Low dose vaccine + BZN	25 µg Tc24-C4 + 5 µg E6020 SE	25 mg/kg orally for 7 days
High dose vaccine + BZN	100 µg Tc24-C4 + 25 µg E6020 SE	25 mg/kg orally for 7 days

All mice were infected with 500 *T. cruzi* H1 by intraperitoneal injection. Treated mice were prime-immunized subcutaneously on day 7 and boosted at day 14 post-infection. BZN was administered by oral administration once daily beginning 7 days post-infection.

### Parasite burden

Beginning at 10 days post-infection blood samples were collected twice weekly to determine the burden parasite by qPCR. Blood and cardiac parasite burdens were determined by quantitative real-time PCR using TaqMan® Fast Advanced Master Mix (Life Technologies) in ViiA 7 RUO (Applied Biosystems) equipment^36^. Briefly, total DNA was isolated from blood and cardiac tissue using a DNEasy blood and tissue kit (Qiagen) and 4 ng of DNA from blood, or 50 ng of DNA from cardiac tissue was used per reaction. The oligonucleotides primers used to amplify the satellite region of *T. cruzi* nuclear DNA were forward 5′ AST CGGCTGATCDTTTTCGA 3′ and reverse 5′ AATTCCTCCAAGCAGCGG ATA 3′. The PCR reaction included a sequence-specific, fluorescently oligonucleotide probe 5′ 6-FAMCACACACTG GACACCAAMGB 3′. Parasite equivalents were calculated based on a standard curve and data was normalized to GAPDH (primers 5′ CAATGTGTCCGTCGTGGATCT 3′ and 5′ GTCCTCAGTGTAGCCCAAGATG 3′, probe 5′ 6-FAM CGTGCCGCCTGGAGAAACCTGCC MGB 3′, Life Technologies).

### Quantification of inflammatory infiltrate cells and cardiac fibrosis

Heart tissue samples were fixed in 10% neutral buffered formalin and routinely processed for paraffin embedding, sectioning and staining with Hematoxylin and Eosin, or Masson’s Trichrome for inflammation infiltrate or fibrosis measurement, respectively. For inflammation analysis, five representative pictures from each slide stained were captured at 10X magnification using and Amscope ME580 bright field microscope. For inflammatory infiltrate cells the images were analyzed using FIJI software (National Institute of Health) based on the number of pixels corresponding to cells nuclei to estimate the number of inflammatory infiltrate cells per area^69^. To quantify fibrosis, five representative pictures from each slide stained were captured at 20X magnification with Micron software in Micromaster microscope and the percentage of the blue-colored area corresponding to collagen also was determined using FIJI software (National Institute of Health).

### Spleen processing and cultures

At day 53, post-infection mice were euthanized and spleens were disrupted mechanically using a 100 µm cell strainer in presence of RPMI supplemented with 10% fetal bovine serum, antibiotics (1X penicillin–streptomycin) and L-Glutamine. Red blood cells were lysed using ammonium-chloride potassium (ACK) lysis buffer. Spleen cells were adjusted in 5 mL of RPMI supplemented and viability determined by acridine orange and propidium iodide (AOPI) live/dead dye using a Cellometer Auto 2000 cell counter (Nexcelom Biosciences, Lawrence, MA). Spleen cells were incubated at 1 × 10^6^ live-cells per well in a round-bottom 96-well plate and stimulated either with 100 µg/mL recombinant Tc24-C4 protein, 5 µg/mL *T. cruzi* parasite lysate, 20 ng/mL PMA-1 mg/mL Ionomycin as a positive control, or media only for 96 h at 37 °C with 5% CO_2_^36^. After incubation, supernatants were obtained from cell cultures by centrifugation at 300 g for 5 min and then aliquoted and frozen until use.

### T cell cytokines analysis by flow cytometry

To determine CD4 + and CD8 + lymphocytes sub-populations in antigen re-stimulated splenocytes were washed twice with RPMI and surface staining was performed using antibodies anti-CD3 fluorescein isothiocyanate (FITC) clone 145-2C11 (eBioscience), anti-CD8 peridinin chlorophyll protein (PerCP-Cy5.5) clone 53–6.7 (BD Bioscience), and anti-CD4 Alexa Fluor® 700 clone RM4-5 (eBioscience). For intracellular cytokine staining, cells were incubated with 4.1 µg/mL brefeldin A for the last four hours of incubation. After surface staining cells were fixed and permeabilized with BD Cytofix/Cytoperm (BD, Bio Sciences) and stained with anti-IFNγ allophycocyanin (APC) clone XMG1.2 (eBioscience)^59^. Samples were acquired on the analyzer LSR Fortessa and at least 100,000 total events were analyzed using FlowJo 8.7 software. Cells were gated on forward and side scatter for lymphocytes, exclusion of viability dye, and singlet populations (Supplementary Fig. 3A–C, respectively). CD3 + (Supplementary Fig. 3D) , CD8 + and CD4 + gates (Supplementary Fig. 3E) were determined based on surface stained cells. Quadrant gating was used to determine the percentage of CD8 + IL-4 + (Supplementary Fig. 3F), CD4 + IL-4 + (Supplementary Fig. 3G), CD4 + IFNγ + (Supplementary Fig. 3H) and CD8 + IFNγ + cells ((Supplementary Fig. 3I). The percentage of antigen specific or total parasite specific cells for each target was calculated by subtracting the percent of media stimulated cells from the percent of antigen-stimulated cells for each mouse. In cases where the antigen stimulated cells was lower than media alone, the percentage is expressed as a negative number.

### Measurement of cytokines

Antigen-specific cytokines release from re-stimulated spleen cells were measured using a multiplexed bead-based assay Milliplex MAP Mouse T_H_17 (Millipore) and Luminex technology. Supernatant IL-10, IL-5, IL-4, IL-6, IL-21, IL-22, IL-23, IL-17A, IFNγ, IL-12p70 and TNF-α cytokines were measured following manufacturer protocol. Assay was adapted in 96-well wall-less plate format^70^. Briefly, 25 µL of the sample was placed in drops over the magnetic plate and incubated overnight at 4 °C with antibody-immobilized beads. The plates were washed 3 times and incubated with detection antibodies by 1 h at room temperature (RT). To reveal the plates were incubated 30 min RT with streptavidin–phycoerythrin as substrate and placed in Luminex Magpix Reader® and data was analyzed with Bio-plex Manager 6.0 and graphed into radar plot using R software.

### Statistical analysis

Parasitemia throughout the experiment was depicted with the area under curve (AUC). Radar plots were created using package radarchart in R interface^71^. For this, cytokines concentrations data was normalized by applying log base 10 transformation. Transformed values were summarized using the function smean.cl.boot from R package HMisc^72^. No detectable values were transformed in zero. Other data from flow cytometry, cardiac burden parasite, inflammatory cells, and percent fibrosis were analyzed with parametrical and non-parametric tests regarding its distribution. Survival data was analyzed using Log-rank (Mantel-Cox) test. Multiple comparisons were performed with a post-hoc Dunn’s test and significant differences were considered with an adjusted *P*-value < 0.05 using Benjamini–Hochberg procedure.

## Supplementary Information

Supplementary Information.
