# Supplementary material for: Vaccine-linked chemotherapy induces IL-17 production and reduces cardiac pathology during acute Trypanosoma cruzi infection
Source: Sci Rep. 2021 Feb 5;11:3222. doi: 10.1038/s41598-021-82930-w (PMC7865072; doi:10.1038/s41598-021-82930-w)
Supplement: Supplementary file 1 — Supplementary Information. [file 41598_2021_82930_MOESM1_ESM.pdf]

Supplementary Materials for

Vaccine-linked chemotherapy induces IL-17 production and reduces cardiac pathology during acute *Trypanosoma cruzi* infection

Julio V. Cruz-Chan[1,5] Liliana E. Villanueva-Lizama [1,5], Leroy Versteeg[1,6], Ashish Damania[1], Maria José Villar[1],Cristina González-López[5] , Brian Keegan[1], Jeroen Pollet[1], Fabian Gusovsky[3], Peter J. Hotez[1,2,4], Maria Elena Bottazzi[1,2], Kathryn M. Jones[1,2*]

[1] Texas Children's Hospital Center for Vaccine Development, Department of Pediatrics, Baylor College of Medicine, Houston, TX

[2]Department of Molecular Virology and Microbiology, Baylor College of Medicine, Houston, TX

[3] Global Health Research, Eisai, Inc., Cambridge, MA, USA

[4] James A. Baker III Institute for Public Policy, Rice University, Houston, TX

[5] Laboratorio de Parasitología, Centro de Investigaciones Regionales "Dr. Hideyo Noguchi", Universidad Autónoma de Yucatán, Mérida, México

[6] Cell Biology and Immunology Group, Wageningen University & Research, De Elst 1, 6708 WD Wageningen, The Netherlands

*Corresponding author: kathrynj@bcm.edu

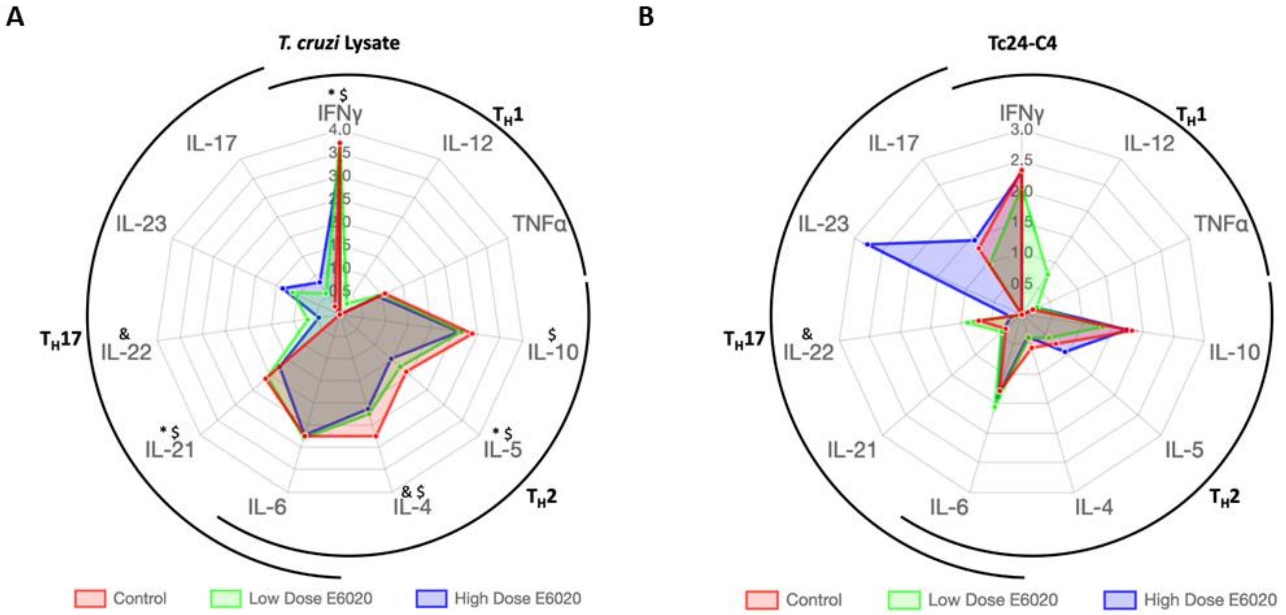

Supplementary Figure 1. Radar plots showing the TH1/TH2/TH17 like cytokine profile upon stimulation with Tc24-C4 or *T. cruzi* Lysate. Cytokines were measured in the supernatant of splenocytes cultures after 96 hours of stimulation. Cytokine profile from mice that receive E6020-SE alone either high (25µg) or low (5µg) doses. *Indicates a significant difference between high-dose and low-dose E6020-SE groups. $Indicates significant difference between high-dose E6020-SE and untreated control group. &Indicates significant difference between Low-dose E6020-SE and untreated control group.

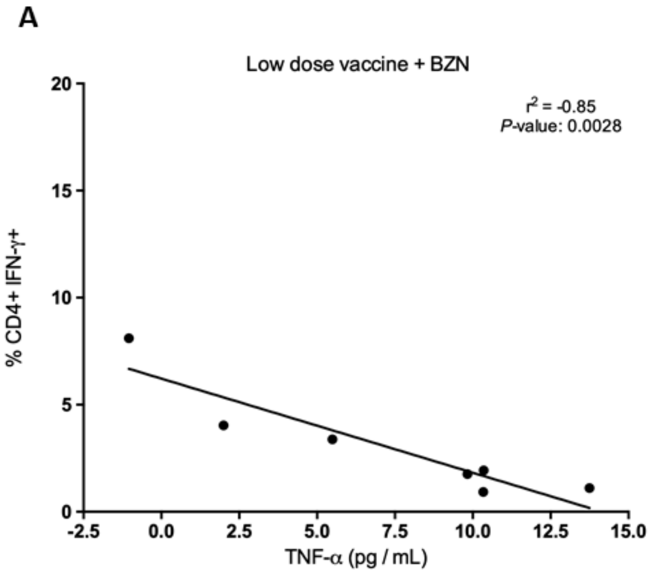

Supplementary Figure 2. Correlation analysis for TNFα and percent CD4+ IFNγ. Mice were treated with either **A)** high or **B)** low vaccine in combination with low-dose of benzidazole at 25mg/kg PO for 1 week starting 7 days post-infection. Levels of TNFα measured by Luminex from supernatant of *T. cruzi* Lysate-stimulated spleen cells were correlated with the percent CD4+ IFNγ from spleen cells *T. cruzi* Lysate stimulated.

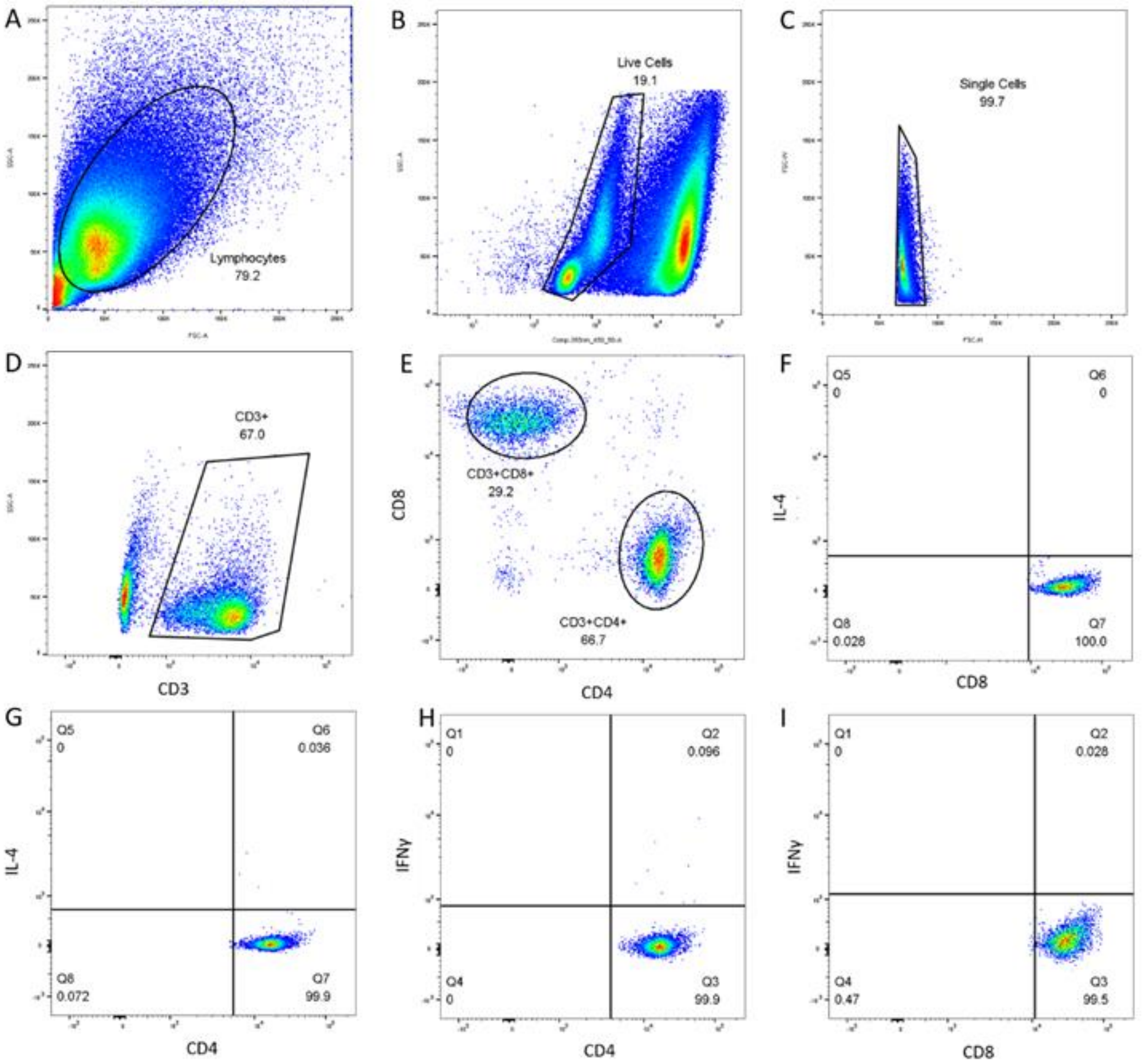

Supplementary Figure 3. Flow cytometry analysis gating strategy. Splenocytes were restimulated in vitro, stained for surface and intracellular markers and acquired for flow cytometry analysis as described. Unstimulated cells stained were used to draw gates for total lymphocytes (A), live cells (B), single cells (C), and CD3+ cells (D). CD3+ cells were used to set gates for CD8+ and CD4+ subpopulations(E). Quadrant gating was used to identify subpopulations of CD8+ IL-4+ (F), CD4+ IL4+ (G), CD4+ IFNγ+(H) and CD8+IFNγ+ (I) cells.

	Infected Untreated	Low dose E6020	High dose E6020	Low dose BZN	High dose BZN	Low dose Vaccine	High dose Vaccine	Low dose Vaccine + BZN	High dose Vaccine + BZN
IL-21	0.4 (0-0.7)	0.4 (0.1-0.7)	0.4 (0.2-0.6)	1 (0.8-1.1)	0	2 (1.5-2.4)	0.5 (0-1)	0.7 (0.4-0.9)	1 (0.9-1.1)
IL-22	0.7 (0-1.4)	0.9 (0.3-1.5)	0.2 (0-0.6)	0.9 (0.4-1.4)	1.5 (0.8-2.1)	1.2 (0.7-1.7)	1.8 (1.5-2)	1.6 (1.1-2)	1.4 (1.1-1.8)
IL-17	1.3 (0.9-1.6)	1 (0.5-1.5)	1.4 (1.2-1.7)	1.2 (1.1-1.4)	1.3 (0.6-2)	1.6 (1.1-2)	1.7 (1.5-1.9)	2 (1.7-2.3)	1.2 (0.9-1.6)
IFNγ	2.4 (2.2-2.6)	2.1 (1.3-2.8)	2.3 (1.7-2.8)	2.2 (1.9-2.6)	1.8 (1.1-2.4)	0.6 (0.4-0.9)	2.3 (2.1-2.6)	2.4 (2.1-2.7)	2.3 (2.1-2.4)
IL-5	0.7 (0.2-1.2)	0.6 (0.2-1)	0.9 (0.5-1.3)	0.2 (0-0.6)	1 (0.5-1.4)	1.1 (0.6-1.6)	1.7 (1.4-1.9)	1.7 (1.5-2)	1.6 (1.3-2)
TNFα	0.2 (0.1-0.3)	0.3 (0.2-0.4)	0.3 (0.2-0.4)	0.6 (0.3-1)	0.4 (0.2-0.6)	1.3 (0.8-1.8)	0.5 (0.4-0.6)	0.8 (0.5-1)	0.7 (0.6-0.9)
IL-6	1.3 (1-1.6)	1.6 (1.1-1.9)	1.4 (1.2-1.6)	0.5 (0.1-0.9)	1.5 (1.1-1.8)	0.7 (0.6-0.9)	2 (1.8-2.1)	1.9 (1.7-2.1)	2.1 (1.9-2.2)
IL-12	0	0.8 (0.7-0.9)	0	1.1 (0.6-1.6)	0.2 (NA-NA)	1.5 (1.1-1.9)	0.3 (NA-NA)	0	0
IL-10	1.8 (1.4-2.2)	1.3 (0.9-1.8)	1.7 (1.6-1.9)	1.1 (0.6-1.5)	0.9 (0.5-1.2)	1.7 (1.2-2)	1.5 (1.4-1.7)	1.6 (1.3-1.9)	1.4 (1.2-1.6)
IL-4	0.6 (0.1-0.9)	0.4 (0.2-0.6)	0.4 (0.2-0.5)	0.3 (0.1-0.4)	0.2 (0.1-0.3)	0.4 (0.3-0.5)	0.8 (0.7-1)	0.7 (0.4-0.9)	0.9 (0.7-1.1)
IL-23	0	0	2.8 (NA-NA)	2.5 (2.1-2.9)	2.4 (NA-NA)	0	1.4 (0.9-1.8)	1.4 (0.6-1.9)	1.6 (0.7-2.2)

Supplementary Table 1. Normalized cytokine values for the radar plot. Values correspond to the mean and 95% confidence interval limits after applying log base 10 transformation. Cytokine levels were measured after 96 hours of stimulation with Tc24-C4 recombinant protein using Luminex. Values from medium-stimulated cells were subtracted from Tc24-C4 stimulated spleen cells. All negatives or no detectable values were transformed into zero values.

	Infected Untreated	Low dose E6020	High dose E6020	Low dose BZN	High dose BZN	Low dose Vaccine	High dose Vaccine	Low dose Vaccine + BZN	High dose Vaccine + BZN
IL-21	2.1 (2-2.2)	2.1 (1.9-2.2)	1.7 (1.5-1.9)	1.8 (1.6-2)	1.6 (1.4-1.9)	1.8 (1.7-2)	1.4 (1.2-1.7)	1.4 (1-1.6)	1.1 (0.5-1.5)
IL-22	0 (0-0)	0.7 (0.2-1.2)	0.5 (0-0.9)	0.7 (0.2-1.1)	2.1 (1.2-2.8)	0 (0-0)	0.4 (0.1-0.7)	1 (0.4-1.5)	0.9 (0.5-1.3)
IL-17	0.2 (NA-NA)	0.5 (0-1)	0.8 (0.4-1.2)	0.7 (0.7-0.8)	1.2 (0.5-1.8)	1 (0.7-1.2)	1.2 (0.9-1.5)	1.1 (0.8-1.4)	1.3 (1.2-1.4)
IFNγ	3.7 (3.4-3.9)	3.7 (3.6-3.8)	3.3 (3.1-3.6)	3.3 (3.2-3.5)	4.5 (3.7-5.2)	3.2 (2.9-3.4)	2.8 (2.6-3.1)	3.4 (2.7-4.1)	3.3 (3.1-3.6)
IL-5	1.9 (1.7-2)	1.7 (1.5-1.9)	1.5 (1.3-1.6)	1.2 (0.9-1.5)	1 (0.4-1.7)	1.9 (1.8-2)	1.7 (1.5-1.8)	1.6 (1.5-1.7)	1.7 (1.5-1.8)
TNFα	1.1 (0.9-1.2)	1 (0.9-1.2)	0.9 (0.7-1.1)	0.7 (0.4-0.9)	1.2 (1.2-1.3)	1 (0.9-1.2)	0.8 (0.7-1)	1.1 (0.9-1.2)	1 (0.9-1.2)
IL-6	2.7 (2.6-2.9)	2.8 (2.7-2.9)	2.7 (2.5-2.9)	2.7 (2.6-2.9)	2.5 (2.3-2.7)	2.6 (2.2-2.9)	2.7 (2.5-2.8)	2.6 (2.5-2.7)	2.6 (2.5-2.7)
IL-12	0 (NA-NA)	0.3 (0.2-0.4)	0 (NA-NA)	0.6 (0.2-1)	0.1 (NA-NA)	0 (NA-NA)	1.1 (NA-NA)	0 (NA-NA)	0 (NA-NA)
IL-10	2.9 (2.8-3)	2.6 (2.5-2.8)	2.6 (2.5-2.7)	2.3 (2.2-2.4)	2.4 (2.2-2.5)	2.1 (1.5-2.6)	2.1 (1.9-2.4)	2.4 (2.2-2.6)	2.3 (2.2-2.3)
IL-4	2.7 (2.7-2.8)	2.2 (2.1-2.4)	2.1 (1.9-2.3)	1.9 (1.6-2.1)	1.5 (1.3-1.7)	2.3 (2.2-2.4)	1.8 (1.6-2.1)	1.8 (1.6-2)	1.9 (1.8-2)
IL-23	0 (NA-NA)	1.1 (1-1.4)	1.4 (0.8-1.9)	1.5 (NA-NA)	0.9 (NA-NA)	2.1 (1.3-2.7)	1.4 (0.9-1.9)	1.5 (0.8-2.1)	1.6 (0.9-2.3)

Supplementary Table 2. Normalized cytokine values for the radar plot. Values correspond to the mean and 95% confidence interval limits after applying log base 10 transformation. Cytokine levels were measured after 96 hours of stimulation with *T. cruzi* Lysate using Luminex. Values from medium-stimulated cells were subtracted from *T. cruzi*-Lysate stimulated spleen cells. All negatives or no detectable values were transformed into zero values.
